# The characteristics of short- and long-term surviving Shiba dogs with chronic enteropathies and the risk factors for poor outcome

**DOI:** 10.1186/1751-0147-55-32

**Published:** 2013-04-17

**Authors:** Hiroki Okanishi, Tadashi Sano, Yoshiki Yamaya, Yumiko Kagawa, Toshihiro Watari

**Affiliations:** 1 Department of Veterinary Medicine, Laboratory of Comprehensive Veterinary Clinical Studies, Faculty of Bioresources, Nihon University, 1866 Kameino, Fujisawa, Kanagawa, 252-0880, Japan; 2 NORTH LAB Inc., Hokkaido, Japan

**Keywords:** Shiba dog, Chronic enteropathies, Risk factors

## Abstract

**Background:**

The objectives of this study were to investigate the differences in the characteristics of short- and long-term surviving dogs, and the factors that predict poor outcome in Shiba dogs with chronic enteropathies (CE).

**Methods:**

A total of 25 Shiba dogs were included in this study, and classified as either short-term (≤6 months) survivors (Ss; n=16) or long-term (>6 months) survivors (Ls; n=9). The clinical and clinicopathological variables, histopathology, response to therapy, and outcomes were investigated between groups. Furthermore, these factors were tested for their ability to predict poor outcome.

**Results:**

All CE dogs were diagnosed as having inflammatory bowel disease (IBD) with lymphocytic-plasmacytic enteritis (LPE). Age and canine inflammatory bowel disease activity index (CIBDAI) were significantly higher in the Ss group than in the Ls group (age: *p* = 0.035, CIBDAI: *p* = 0.018), as determined via univariate logistic regression analysis. According to receiver operator characteristic (ROC) curve analysis, the best predictors of poor outcome were age and CIBDAI, with the cutoffs determined as 7 years and 9 points, respectively. The majority of the cases (84%) responded to initial treatment; in particular, 75% of dogs in Ss group responded to therapy. The time to response (days) to the initial treatment in the Ss group (median 42.5 days, range: 20-91 days) was significantly shorter than that of the Ls group (median 285 days, range: 196-1026 days). Approximately half (55.5%) of the dogs in the Ls group died due to relapse of CE.

**Conclusions:**

This study suggested that there is a high risk of early mortality in Shiba dogs with CE, particularly if the dogs are older (>7 years) and have a high CIBDAI score (>9 points). There appears to be a possibility of early mortality even if the initial treatment was efficacious. Furthermore, Shiba dogs with CE that become less responsive to initial therapy in the short-term (approximately 3 months) are more likely to have an early mortality. Thus, it is necessary to follow-up Shiba dogs with CE in the long-term, as approximately half of the long-term survivors eventually died due to a relapse of the signs.

## Background

Chronic enteropathies (CE) are commonly encountered in dogs, and categorized into inflammatory bowel disease (IBD), antibiotic-responsive diarrhea (ARD), and food-responsive diarrhea (FRD), according to the response to treatment [1]. Similar to human IBD, the molecular basis for the pathogenesis of this disease is unclear, although it has been suggested that the disease occurs in genetically susceptible hosts as a consequence of a dysregulated response of the mucosal immune system toward commensal enteric flora and dietary components [2,3]. There are several reports on dogs with specific types of CE (e.g., German shepherds [4] and Basenjis [5]), including Shiba dogs [6]. Shiba dogs are known to be affected by immunological diseases, such as atopic dermatitis [7], and it is likely that altered immune reactions to enteric bacteria and dietary components may be occurring in their gastrointestinal tracts [8]. Furthermore, Shiba dogs with CE have poor responses to therapy compared to other breeds, and it has been previously reported that they have a six-month survival rate of approximately 50% [6]. To the best of our knowledge, there are no previous reports on the risk factors for poor outcomes in this breed with CE. Furthermore, while many Shiba dogs with CE die in the short-term, there are some dogs that manage to have long-term survival time.

Thus, the purpose of the present retrospective study was to determine the differences in clinical and laboratory features, histopathology, response to therapy, and outcomes between short- and long-term surviving Shiba dogs with CE. Furthermore, we aimed to investigate the risk factors that predict poor outcomes in Shiba dogs with CE.

## Materials and methods

The hospital records of Shiba dogs with CE that were diagnosed at the Animal Medical Center of Nihon University during a six-year period (2005-2011) were retrospectively reviewed. The inclusion criteria for this study were histopathological evidence of gastric and small intestinal inflammation on endoscopic biopsies; chronic gastrointestinal signs, such as vomiting, diarrhea, and weight loss, consistent with CE, for at least 3 weeks in duration, and the exclusion of all other possible causes of signs. A minimal diagnostic evaluation to exclude other diseases was performed, which consisted of complete blood cell (CBC) count, serum chemistry, fecal examination, survey radiography, and abdominal ultrasonography. Cases were excluded if underlying or concurrent disorders other than CE were confirmed. Shiba dogs were classified into either the short- or long-term survival groups (Ss and Ls groups, respectively) at 6 months after the first diagnosis.

Sex, age, and body weight were compared in both the Ss and Ls groups. All dogs were given a clinical score using the canine inflammatory bowel disease activity index (CIBDAI) scoring system [9], and the scores were compared between both groups. The scoring criteria included attitude/activity, appetite, vomiting, stool consistency, stool frequency, and weight loss. Using the CIBDAI, six prominent gastrointestinal signs were scored from 0 to 3 based on the magnitude of their changes.

Hematological variables, including packed cell volume (PCV), blood platelet (PLT) count, white blood cell (WBC) count, and neutrophil, lymphocyte, monocyte, and eosinophil counts, were compared between the two groups. Blood coagulation, namely levels of antithrombin (AT) activation, prothrombin time (PT), activated partial thromboplastin time (APTT), and fibrinogen (Fib), was also compared. Additionally, the following biochemical variables were compared: blood urea nitrogen (BUN), creatinine (CRE), alkaline phosphatase (ALP), alanine aminotransferase (ALT), calcium (Ca), inorganic phosphorus (IP), glucose (Glu), sodium (Na), potassium (K), chloride (Cl), albumin (ALB), globulin (Glob), total cholesterol (T. Chol), total protein (TP), serum protein fraction (α1, α2, β, and γ), and C-reactive protein (CRP).

Endoscopical examinations were performed in all dogs. The histopathological severity of the biopsy samples was scored by an American College of Veterinary Pathologists (ACVP) board-certified pathologist as normal (score 0), mild (score 1), moderate (score 2), or severe (score 3). The scores were based on a comprehensive analysis of inflammatory cell infiltration and structural changes (i.e., stunting and fusion of villi, lacteal dilation, epithelial injury, and crypt distension) in the mucosa of gastrointestinal tract.

At the local hospitals, all dogs received antibacterial and dietary therapy consisting of an antigen-restricted diet, a hydrolyzed diet, or a highly digestible diet (>3 weeks); however, none of the dogs responded to these treatments. At our hospital, all dogs were treated with metronidazole (10 mg/kg/day, PO, b.i.d., >2 weeks). Additionally, the dogs that were fed with the highly digestible diet at the local hospitals were switched to an elimination diet (>3 weeks), as part of their initial treatment for CE. Furthermore, all cases were treated with either 1 mg/kg/day (Ss: 6 and Ls: 8 dogs) or 2 mg/kg/day (Ss: 10, and Ls: 1 dogs) of prednisone, and these doses were gradually increased to a maximum of 4 mg/kg/day depending on the therapeutic response. Dogs that did not respond to steroid therapy received other immunosuppressive agents, cyclosporine (5-10 mg/kg/day) or azathioprine (2 mg/kg/day). Treatment responsiveness was defined according to the following definitions: ‘good’ indicated a satisfactory responsiveness with a complete disappearance of signs, ‘partial’ indicated partial responsiveness with an incomplete disappearance of signs, and ‘poor’ indicated unresponsiveness. Responsiveness to the initial medication was scored from 0 to 2 (i.e., 0 = poor, 1 = partial; and 2 = good) in both groups. Additionally, the time to response (days) to the initial treatment were compared between the two groups.

The outcomes of both the Ss and Ls groups were compared, specifically the number of days from becoming unresponsive to treatment until mortality, survival time (days), and mortality. Follow-up information was obtained from local hospitals for long survival cases. Risk factors for poor outcome were determined from the clinical and clinicopathological variables, histopathology, response to therapy, and outcomes between the two groups.

### Statistical analysis

Data that were not normally distributed were reported as medians (ranges). Categorical data were presented as either percentages or ratios. The Fisher’s exact test was used to compare categorical data. The Mann-Whitney U test was used to compare numerical data between the Ss and Ls groups. Kaplan-Meier survival curves and log-rank testing were used to analyze survival data. Correlations were evaluated with the Spearman’s rank correlation test. Odds ratios (OR) were determined using logistic regression models with a single predictor. Multivariate logistic regression analysis was attempted; however, the sample size was too small. Univariate logistic regression analyses were performed to determine the risk factors for poor outcome. Receiver operator characteristic (ROC) curve analyses were used for the determination of cutoff values for age and CIBDAI. Statistical significance was set at *p* < 0.05. All statistical analyses were performed using commercially-available statistical software systems (Prism 5 for Mac OS, GraphPad Software Inc., San Diego, CA, USA, SigmaPlot 12, SYSTAT Software Inc., San Jose, CA, USA).

## Results

### Sex, age, and body weight

A total of 25 dogs were included in this study: 16 in the Ss group and nine in the Ls group. All CE dogs failed to respond to dietary and antibiotic therapy, and were diagnosed as having IBD with lymphocytic-plasmacytic enteritis (LPE).The characteristics of each group are presented in Table 1. There were nine males, four of which were neutered, and seven females, four of which were spayed in the Ss group. In the Ls group, there were six males, two of which were neutered, and three females, all of which were spayed. No statistically significant differences were found in the sex distribution between the two groups (*p* = 0.6913).

**Table 1:** Characteristics and clinical scores in the short-term (Ss) and long-term (Ls) groups

	**Ss**	**Range**	**n**	**Ls**	**Range**	**N**	***P***
**Sex (Male : Female)**	9 : 7	-	16	6 : 3	-	9	0.6913
**Age (years)**	7.5	3-13	16	5	1-10	9	0.0462
**Body weight (kg)**	10.5	4-17	16	10	4.8-14	9	0.9321
**CIBDAI (score)**	12	4-17	16	7	4-13	9	0.0131
**Attitude/Activity**	2	0-3	16	0	0-2	9	0.0364
**Appetite**	2	0-3	16	0	0-3	9	0.01
**Vomiting**	0	0-3	16	2	0-3	9	0.229
**Stool consistency**	3	2-3	16	3	0-3	9	0.156
**Stool frequency**	1	0-3	16	0	0-2	9	0.1047
**Weight loss**	3	1-3	16	2	0-3	9	0.0337

CIBDAI, canine inflammatory bowel disease activity index.

The median age of all dogs was 7 years (mean 8.3). In the Ss group, the median age was 7.5 years (mean: 7.6, range: 3-13 years), and in the Ls group, the median age was 5 years (mean: 5.2, range: 1-10 years). The age of the Ss group was significantly higher than that of the Ls group (*p* = 0.0462).

The median body weight of the Ss group was 10.5 kg (range: 4-17 kg), and of the Ls group was 10 kg (range: 4.8-14 kg). No statistically significant differences were seen in the body weight between the two groups (*p* = 0.9321).

### Clinical signs and scores

The clinical signs and CIBDAI scores of the each group are presented in Table 1. All dogs presented with small bowel diarrhea. In the Ss group, the median of the CIBDAI score was 12 points (range: 4-17 points), whereas in the Ls group, it was 7 points (range: 4-13 points). The CIBDAI score of the Ss group was significantly higher than that of the Ls group (*p* = 0.0131). In particular, the median scores of the attitude/activity, appetite, and weight loss categories were significantly higher in the Ss group when compared to those of the Ls group (attitude/activity: *p* = 0.0364, appetite: *p* = 0.01, weight loss: *p* = 0.0337).

### Laboratory findings

Hematology testing was performed for all dogs, but no statistically significant differences were noted between the Ss and Ls groups. Additionally, no statistically significant differences were present between the two groups with respect to blood coagulation.

In regards to biochemical findings, significant differences were observed between the two groups in TP, CRE, and CRP concentrations (Table 2). The median of the TP concentration in the Ss group (5.4 g/dl, range: 3.6-7.3) was significantly lower than that of the Ls group (6.2 g/dl, range: 4.8-7.6) (*p* = 0.0173). Hypoproteinaemia (TP <5.2 g/dl) was present in six dogs (37.5%) in the Ss group, five of which had low albumin and normal globulin levels, and one of which had low albumin and globulin levels. Hypoproteinaemia was present in only one dog (11.1%) in the Ls group, which had a low total serum protein and albumin levels and normal globulin levels. The median CRE in Ss the group (0.8 mg/dl, range: 0.4-1.1) was significantly lower than that of the Ls group (1 mg/dl, range: 0.4-1.7) (*p* = 0.0194). The CRE concentration was below the reference range (0.5-1.8 mg/dl) in two dogs (12.5%) in the Ss group, and only one dog (11.1%) in the Ls group. In the Ss group, the median CRP was 1.7 (range: 0.05-12), whereas in the Ls group, it was 0.15 (range: 0-2.5). CRP was significantly higher in the Ss group than in the Ls group (*p* = 0.0135). Elevated CRP was present in 10 dogs (62.5%) in the Ss group, and two dogs (22.2%) in the Ls group.

**Table 2:** Comparisons of laboratory findings in the short-term (Ss) and long-term (Ls) groups

	**Ss**	**Range**	**n**	**Ls**	**Range**	**n**	***P***
**WBC (/μl)**	17750	11300-50700	16	16000	7200-21900	9	0.3648
**PLT (/μl)**	420000	60300-611000	16	332	153-531	9	0.1486
**PCV (%)**	37	27.5-49.0	16	38	31.0-46.0	9	0.3071
**AT (%)**	87	68-134	14	99	67-109	7	0.5504
**ALB (g/dl)**	1.65	0.7-3.1	16	2.3	1.6-3.5	9	0.1124
**TP (g/dl)**	5.4	3.2-7.3	16	6.2	4.8-7.6	9	0.0173
**Glob (g/dl)**	3.2	2.6-4.5	16	3.6	3-4.8	9	0.0887
**CRE (mg/dl)**	0.8	0.3-1.1	16	1.0	0.4-1.7	9	0.0194
**T.Chol (mg/dl)**	109.5	54-229	16	98	69-238	9	0.8207
**Ca (mg/dl)**	8.9	7-9.9	15	9.4	8.5-10	7	0.1116
**CRP (mg/dl)**	1.7	0.05-12	16	0.15	0-2.5	9	0.0135

WBC, white blood cell; PLT, blood platelet; PCV, packed cell volume; AT, antithrombin; ALB, albumin.

TP, total protein; Glob, globulin; CRE, creatinine; T. Chol, total cholesterol; Ca, calcium; CRP, C-reactive protein.

### Histopathological scores

All biopsies were re-evaluated by an American College of Veterinary Pathologists (ACVP) board-certified pathologist in a blinded fashion. Endoscopy was performed in all dogs, and the stomach and duodenum were histopathologically scored (Table 3). Biopsy samples revealed inflammation with infiltration of lymphocytes and plasma cells. A significant difference was seen between the Ss and Ls groups in the histopathological score of the duodenum. In the duodenum, the median score in Ss group (3, range: 2-3, mean: 2.8) was significantly higher than that of the Ls group (3, range: 1-3, mean: 2.3) (*p* = 0.0231). There were 14 (87.5%) dogs in Ss group and six (66.6%) dogs in Ls group that were assessed as severe (Score 3).

**Table 3:** Comparisons of the histopathological scores, response to therapy, and outcome in the short-term (Ss) and long-term (Ls) groups

	**Ss**	**Range**	**n**	**Ls**	**Range**	**n**	***P***
**Histopathological score**							
**Stomach**	0	0-3	12	0	0-3	9	0.9405
**Duodenum**	3	2-3	16	3	1-3	9	0.0231
**Therapy**							
**Treatment score**	1	0-2	16	2	2	9	0.0079
**Time to response (days)**	42.5	20-91	12	285	196-1026	5	0.0019
**Number of days between treatment unresponsiveness and death**	19.5	0-90	12	151	35-218	5	0.0131
**Outcome**							
**survival (days)**	73	26-171	16	800	231-2204	9	<0.0001
**Mortality (%)**	100	-	16	55.5	-	9	0.01

In the stomach, no statistically significant differences were found between the two groups (*p* = 0.9405). Indeed, many cases (Ss: 12 dogs, 75%; Ls: 6 dogs, 66.6%) were histopathologically assessed as normal (Score 0). There were only two dogs classified as severe (Score 3), with a dog in each group (Ss: 11.1%, Ls: 6.2%).

### Responsiveness to treatment

The total number of cases that were administrated a prednisone dose greater than 2 mg/kg/day were as follows: 11 out of 16 cases in the Ss group and one out of nine cases in the Ls group. There were significantly more dogs that were administered a prednisone dose greater than 2 mg/kg/day in the Ss group than Ls group (*p* = 0.0112). Furthermore, the prednisone dose could only be decreased in three out of 16 dogs in Ss group and seven out of nine dogs in the Ls group. The number of dogs in the Ls group that had their prednisone dose lowered was significantly greater than that of the Ss group (*p* = 0.0090). Only two dogs in the Ls group were completely weaned off prednisone. The other seven dogs in the Ls group were maintained on anti-inflammatory doses of 0.5 mg/kg/day (n=5) or 1 mg/kg/day (n=2) of prednisone over the long-term. Twenty-one out of 25 dogs (84%) responded to initial treatment. Of these, seven dogs (43.8%) had ‘good’ responsiveness, five dogs (31.2%) had ‘partial’ responsiveness, and four dogs (25%) had ‘poor’ responsiveness in the Ss group. In the Ls group, all nine dogs (100%) had ‘good’ responsiveness. The median score of responsiveness to initial treatment in the Ss group (score: 1, range: 0-2) was significantly lower than that of the Ls group (score: 2, in all dogs) (*p* = 0.0079).

The response time (days) to initial treatment could not be determined in the cases (Ls: n=4) that remained responsive to treatment and did not become unresponsiveness. Furthermore, the cases (Ss: n=4) with poor responsiveness could not be included, as they did not respond to treatment at all (i.e., there was not a single day that they had responsiveness). Therefore, these cases were excluded. Of the cases with ‘good’ or ‘partial’ responsiveness, the median number of days to a response to initial treatment was 42.5 days in the Ss group (range: 20-91) and 285 days in the Ls group (range: 196-1026). The time to a response in the Ss group was significantly shorter than that of the Ls group (*p* = 0.0019). These data are presented in Table 3.

Cyclosporine and azathioprine was administered to dogs that had a poor responsiveness to steroid therapy. A total of 12 dogs were given cyclosporine, specifically ten (62.5%) in the Ss group and two (22.2%) in the Ls group. Additionally, only one dog in the Ss group was given azathioprine. However, no dogs were responsive to these immunosuppressive agents.

### Outcome

In the analysis of the number of days from unresponsiveness to treatment until death, cases that remained responsive to treatment (Ls: n=4) and cases with ‘poor’ responsiveness (Ss: n=4) were excluded. The median number of days from unresponsiveness to treatment until death in the Ss group was 19.5 days (range: 0-90), and in the Ls group, was 151 days (range: 35-218), which was significantly longer than that of the Ss group (*p* = 0.0131) (Table 3).

**Figure 1:**
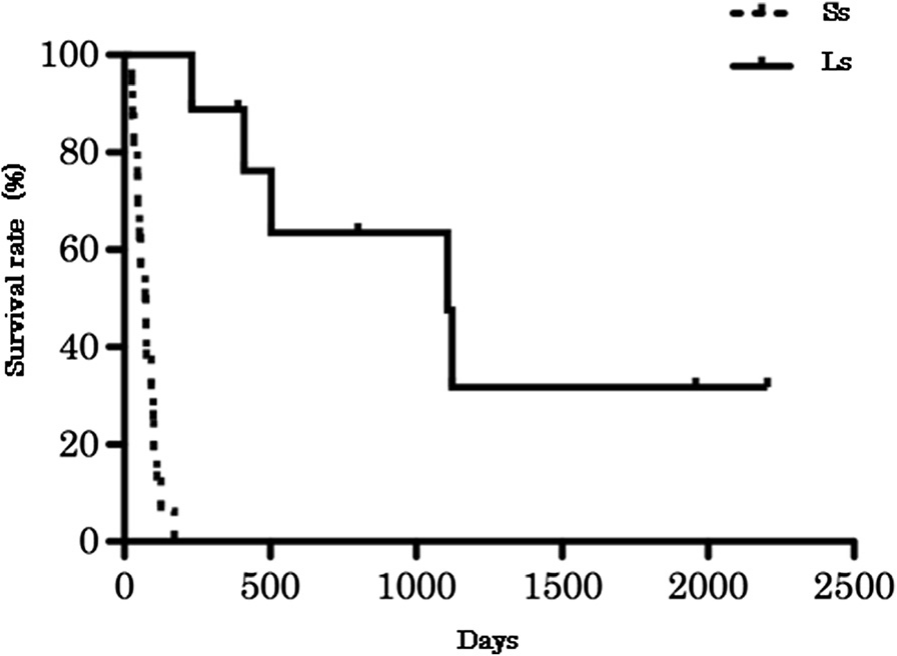
**The survival rates of the short-term (Ss) and long-term (Ls) groups, as represented by the Kaplan-Meier survival curves.** The median number of survival days in the Ss group (73 days, range: 26-171) was significantly lower than that in the Ls group (800 days, range: 231-2204) (log-rank test p < 0.0001, Ss: n = 16, Ls: n = 9).

The median number of survival days in the Ss group (73 days, range: 26-171) was significantly lower than that of the Ls group (800 days, range: 231-2204) (*p* < 0.0001) (Figure 1). The median number of survival days in all Shiba dogs was 101 days. The survival rates for 6 months, 1 year, 3 years, and 5 years were 36% (9/25), 32% (8/25), 16% (4/25), and 8% (2/25), respectively, in all dogs.

The mortality rate of all dogs was 84% (n=21). Of these dogs, 16 dogs (100%) were included in the Ss group, and five dogs (55.5%) were in the Ls group. The Ss group had a significantly greater mortality rate than the Ls group (*p* = 0.01) (Table 3). All dogs died from diseases related to CE. The main cause of death was debilitation due to malabsorption (18/21, 85.7%) and gastrointestinal bleeding (3/21, 14.3%).

### The correlations between characteristics, clinical signs, laboratory findings, response to treatment, and outcome

The correlations between age, CIBDAI, TP, CRP, time (days) to a response to initial treatment, number of days from unresponsive to treatment until mortality, and survival time (days) of all Shiba dogs were assessed (Table 4). In all Shiba dogs, significant associations were found between survival time and age (r_s_ = -0.5240, p = 0.0072), CRP (r_s_ = -0.56, p = 0.0036), TP (r_s_ = 0.4462, p = 0.0254), and number of days from unresponsive to treatment until mortality (r_s_ = 0.6426, p = 0.0054). Additionally, significant correlations were seen between CIBDAI and CRP (r_s_ = 0.5161, p = 0.0083), the time (days) to a response to initial treatment and number of days from unresponsive to treatment until mortality (r_s_ = 0.5298, p = 0.0287).

**Table 4:** Correlations between characteristics, clinical signs, laboratory findings, response to treatment, and outcome

	**r**	**n**	***P***
**Survival (days), Age**	-0.524	25	0.0072
**Survival (days), CIBDAI**	-0.3439	25	0.0999
**Survival (days), CRP**	-0.56	25	0.0036
**Survival (days), TP**	0.446	25	0.0254
**Survival (days), Days kept to response**	0.6246	17	0.0054
**CIBDAI, CRP**	0.5161	25	0.0083
**CIBDAI, Days kept to response**	-0.4528	17	0.068
**CRP, Days kept to response**	-0.2641	17	0.305
**Time to response (days), Number of days between treatment unresponsiveness and death**	0.5298	17	0.0287

CIBDAI, canine inflammatory bowel disease activity index; CRP, C-reactive protein; TP, total protein.

### Risk factors for poor outcome

**Figure 2:**
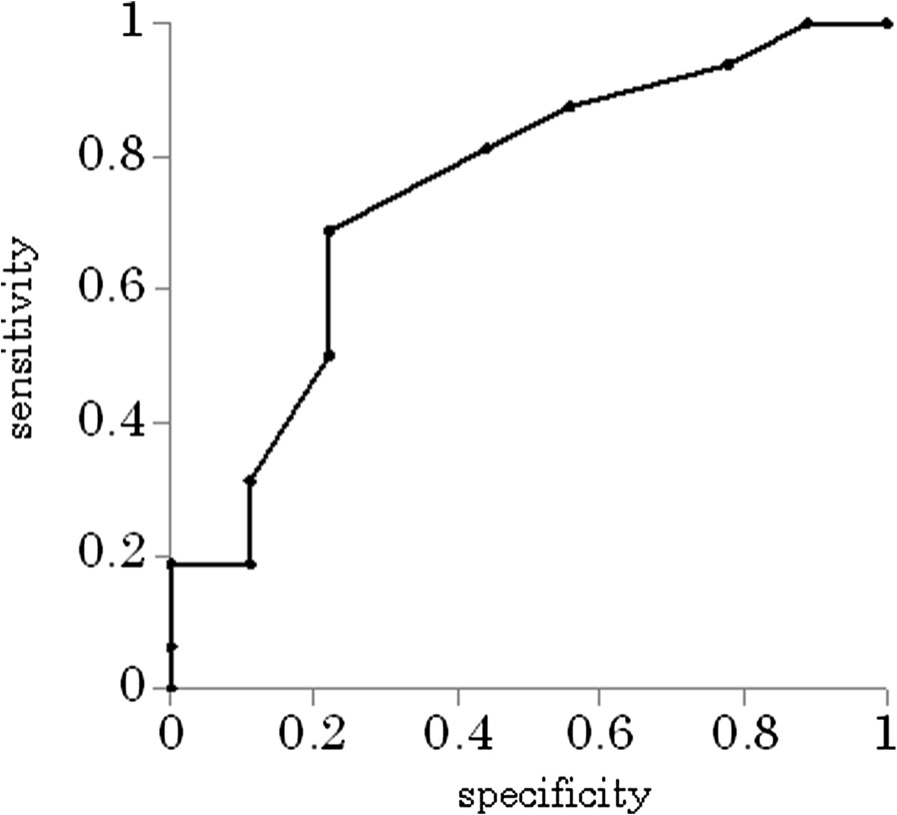
**The cutoff value for age, as determined by receiver operator characteristic (ROC) curves.** A cutoff value of 7 years was shown to be the best predictor for poor outcome, with a sensitivity of 0.7, a specificity of 0.78, and area under the curve (AUC) of 0.81 (*p* = 0.035, odds ratio (OR): 7.7, 95% confidence interval (CI): 1.1-51.2).

**Figure 3:**
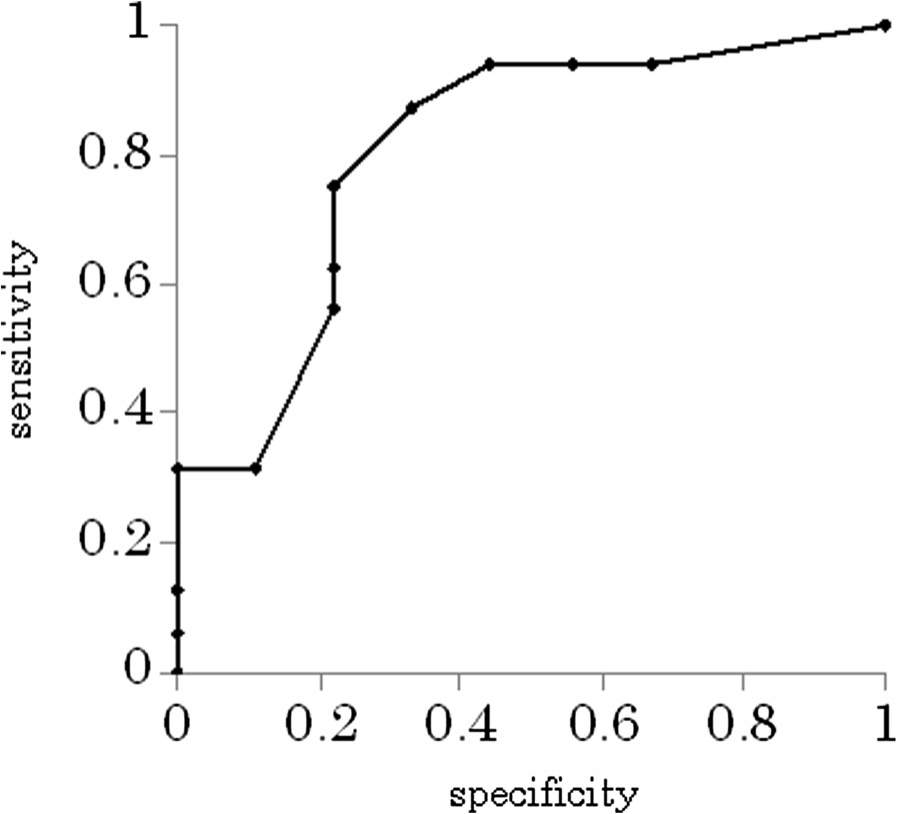
**The cutoff value for CIBDAI, as determined by receiver operator characteristic (ROC) curves.** A cutoff value of 9 was shown to be the best predictor for poor outcome, with a sensitivity of 0.88, a specificity of 0.68, and area under the curve (AUC) of 0.75 (*p* = 0.018, odds ratio (OR): 1.45, 95% confidence interval (CI): 1.1 -1.9). CIBDAI, canine inflammatory bowel disease activity index.

Age, CIBDAI, CRP, the score of responsiveness to initial treatment, and the time (days) to a response to initial treatment were analyzed as risk factors for poor outcome. Univariate logistic regression analyses revealed that age was a risk factor for poor outcome (*p* = 0.035, OR: 7.7, CI 95%: 1.1-51.2). For age, the cutoff value of 7 years was shown to be the best predictor for poor outcome, with a sensitivity of 0.7, a specificity of 0.78, and area under the curve (AUC) of 0.81 (Figure 2). CIBDAI was also a risk factor for poor outcome (*p* = 0.018, OR: 1.45, CI 95%: 1.1 -1.9). For CIBDAI, a cutoff value of 9 was shown to be the best predictor for poor outcome, with a sensitivity of 0.88, a specificity of 0.68, and AUC of 0.75 (Figure 3). No other factors assessed in this study were determined to be predictors for poor outcome.

## Discussion

In a previous study on Shiba dogs with CE, the median age was 4.9 years [6], which was almost comparable to the ages reported in other retrospective studies of dogs with IBD and CE [10-12]. Similarly, in our study, the median age of the Ls group was 5 years (mean 5.2). However, in the Ss group, the median age was 7.5 years (mean 7.6), and in all Shiba dogs, the median age was 7 years. Additionally, a negative correlation was observed between survival time and age in the analysis of all dogs. In a previous study, dogs requiring steroid therapy were middle-aged and older (mean: 6.5 years) [10]. Older Shiba dogs with CE may have different pathological mechanisms and poor responsiveness to steroid therapy compared with those of younger dogs. Thus, aging Shiba dogs with CE require attention because they may have a shorter survival time. Furthermore, it is predicted that dogs aged >7 years have a higher risk of poor outcome.

There were no previous reports assessing the CIBDAI in Shiba dogs with CE; however, there was one report that found anorexia and a decrease in activity were more likely to be present in the non-survivor than in the survivor Shiba group [6]. Another study found that anorexia and severe weight loss in dogs with LPE, including many Shiba dogs, were significantly more evident in non-survivors than in survivors, and suggested that anorexia could be a risk factor of poor outcome [8]. In our study, we also found that a decrease in activity and weight loss were significantly more evident in the non-survivor than in the survivor Shiba groups; however, no differences were observed in anorexia. These differences may be because anorexia is a subjective index. The CIBDAI score in the Ss group was significantly higher than that in the Ls group. In a previous study, dogs with CE were reported to have a higher CIBDAI score (score >8), which was a risk factor for poor outcome. However, in this study, the canine chronic enteropathy clinical activity index (CCECAI; includes assessments of serum albumin, peripheral edema and ascites, and pruritus) was much more powerful in accurately predicting poor outcome than CIBDAI. In our study, a score >9 was a risk factor for poor outcome, and thus, CIBDAI appears to be an important tool for predicting the outcome in Shiba dogs with CE. Unfortunately, we could not determine the CCECAI because our report was a retrospective study. Further studies may be needed to investigate whether CCECAI is also a predictor.

A previous report on Shiba dogs found that TP levels were significantly lower in non-survivors than in survivors [6]. Moreover, a study on dogs with LPE corroborated this report, and found hypoproteinaemia in 15 (93.8%) out of 16 dogs in the non-survivors group, suggesting that TP may be a prognostic factor of poor outcome [8]. Our study found that hypoproteinaemia was present in only 37.5% of dogs in the Ss group. Despite this, the median TP in the Ss group was significantly lower than that in the Ls group. We also found that TP correlated with survival time, where dogs with lower TP levels had shorter survival times. Based on these findings, TP may be a difficult variable to use as a risk factor of poor outcome in CE Shiba dogs. Regardless, dogs with low TP levels should be monitored.

Previous studies have assessed CRP levels in dogs with CE with no correlations observed between CRP and outcome or CIBDAI [6,10,13,14]. In our study, it was found that CRP in the Ss group was significantly higher than that in the Ls group. Additionally, an association was found between CRP and survival time (a negative correlation) or CIBDAI (a positive correlation) in all Shiba dogs. However, CRP was difficult to use as a risk factor of poor outcome in Shiba dogs with CE in univariate logistic regression analyses. A severe inflammatory reaction can induce serious clinical signs and have a bad prognosis in Shiba dogs with CE. Therefore, dogs with elevated CRP require attention with respect to outcome. CRP might be a useful risk factor of poor outcome in Shiba dogs with CE, if the study number had been large enough.

Several reports have previously suggested that no associations can be found between histopathological changes with clinical severity or outcome [6,10,13,14]. In a previous study on Shiba dogs with CE, it was reported that 75% of the dogs in the Shiba group were judged as having severe lesions in the duodenum by several pathologists [6]. In our study, 87.5% and 66.6% of duodenal lesions in the Ss and Ls groups, respectively, were assessed as severe by a single pathologist, and no statistical difference was seen in the percentages between the groups. Thus, the histological severity of the duodenum does not appear to account for the poor prognosis of LPE in Shiba dogs. However, Shiba dogs with CE and severe duodenal lesions require attention, as the median histopathological score in the cases with a short-term survival time was significantly higher than those with a long-term survival. Interestingly, the stomach was histologically normal in most dogs with short- and long-term survival. Severe changes in the duodenum and no changes in the stomach may be one of the clinical features of Shiba dogs with CE. It should be noted that this study did not use the recently published World Small Animal Veterinary Association (WSAVA) histopathological standards due to its retrospective nature. Further studies are warranted to determine the histopathological changes with this classification system in Shiba dogs with CE.

Many of the cases (10/16 cases) in the Ss group had to be initiated on a prednisone dose of 2 mg/kg/day at first administration. However, only one case in the Ls required a starting prednisone dose of 2 mg/kg/day. The reason for this was because Ss dogs had more severe scores for the clinical signs and laboratory findings than Ls dogs (milder scores). There was a significantly greater number of cases administrated a prednisone dose of >2 mg/kg in the Ss versus the Ls group. This suggests that the dogs in Ss group required higher doses of prednisone, and may be explained by the more severe score in the clinical signs and laboratory findings compared to the Ls group. Conversely, all of the dogs in the Ls group were either completely weaned off prednisone or satisfactorily maintained on anti-inflammatory doses of 0.5 mg/kg/day or 1 mg/kg/day of prednisone over the long-term. Thus, long-term survival of Shiba dogs with CE could be maintained with a relatively low dose of prednisone.

The majority of the cases (84%) responded partially or satisfactory to the initial treatment. Specifically, 75% of the dogs with short-term survival and all of the dogs with long-term survival responded to the therapy. However, the score of responsiveness to initial treatment in the Ss group was significantly lower than that of the Ls group. There was one previous study on the responsiveness to medication in dogs with LPE [8]. In the study, only 12.5% of the dogs with short-term survival time and 87.5% with long-term survival responded to the therapy, and therefore, the response to initial treatment may be a risk factor of poor outcome. Some reasons for the differences between our study and their report are as follows: (1) our study was conducted on Shiba dogs only; (2) the treatment protocols were different; and (3) the assessments of the response to therapy were subjective. Based on our results, while Shiba dogs with CE are more likely to have some response to initial treatment, but it is also likely that they will have a poor prognosis in the near future. Thus, the response to initial treatment may be difficult to use as a risk factor of poor outcome in Shiba dogs with CE.

The time (days) to a response to initial treatment in the Ss group (42.5 days, range: 20-91 days) was significantly shorter than that in the Ls group (285 days, range: 196-1026 days). Furthermore, the number of days between treatment unresponsiveness until death in the Ss group was significantly lower than that in the Ls group. A positive correlation was also found between the time (days) to a response to initial treatment and survival duration. These results indicate that Shiba dogs with CE that became less responsive to therapy within a short period of time (about 3 months) are more likely to die in the short-term, even with subsequent therapy. The reasons for the differences in the response time (days) to the initial treatment between the two groups are unknown. However, one clinical study on steroid resistant IBD cases reported that Nuclear factor-kappa B, a transcriptional factor is activated in the intestinal mucosa, which leads to a decrease in the number of glucocorticoid receptors, and thereby, a decrease in their anti-inflammatory effects [15]. In another study on dogs, it was reported that low serum cobalamin concentrations are associated with refractoriness to treatment in dogs with CE [13]. Moreover, another study reported that increases in P-glycoprotein, a drug-efflux pump, within the intestinal mucosa are associated with responses to prednisone in IBD dogs [16]. Taken together, the findings of these studies suggest that the differences in the pathological condition may be, in part, due to difference in the abovementioned factors, and thereby, response time (days) to treatment. However, we did not assess these factors in the present study. Thus, further studies are warranted to investigate the differences between short- and long-term survival in Shiba dogs with CE. It should be noted that the cases that remained responsive to treatment (Ls: n=4) or had ‘poor’ responsiveness (Ss: n=4) were excluded in the analysis of the response time (days) to initial treatment. If these cases were included the analysis, the result may be different.

Cyclosporine has been reported to be an effective drug in dogs with steroid refractory IBD [17]. However, in the present study, no dogs responded to cyclosporine. This may be because the intestinal malabsorption of cyclosporine was hindered by the severe enteropathy; however, we cannot confirm the exact reason, as we did not assess blood levels of cyclosporine in this study. Further studies may be needed to investigate about cyclosporine. Cyclosporine has little curative effect in Shiba dogs with CE, and other treatments may be needed in future. Additionally, it might be necessary to investigate the efficacy of cyclosporine for Shiba dogs with CE at early stage, before the enteritis is too severe. This study is limited by the differences between Ss and Ls groups in (1) the doses of prednisone, particularly the starting doses, and (2) the number of dogs administered immunosuppressive agents (i.e., cyclosporine and azathioprine). However, these differences are due to the retrospective nature of the study. Further studies are needed to investigate the differences between long- and short-term survival of Shiba dogs with CE treated with the same therapies and doses

The median survival duration in the Ss and Ls groups were 73 and 800 days (all Shiba dogs: 101 days), respectively. The survival rates of all Shiba dogs at 6 months, 1 year, 3 years, and 5 years were 36%, 32%, 16%, and 8%, respectively. In a previous study on Shiba dogs, the median survival was 74 days, and the survival rates at 6 months and 1 year were 46% and 31%, respectively [6]. In the other reports on dogs with IBD and LPE, which included several breeds, the survival rates at 6 months were 96% and 74%, respectively [8,12]. Compared to these reports, our findings strongly suggest a poor outcome for Shiba dogs with CE. Additionally, dogs that survive >6 months are more likely to remain alive after >1 year, as no significant differences have been noted between 6 months and 1 year in the survival rate. However, about half (55.5%) of the dogs that survived >6 months (Ls group) died due to a relapse of CE. This indicates that a long-term follow-up is necessary in Shiba dogs with CE, even if the response to treatment is satisfactory. Future studies on Shiba dogs with CE may aid in elucidating the pathology of CE, as well as effective therapy.

## Conclusions

The findings of the present study suggest that Shiba dogs with CE have a high risk of early mortality (≤6 months), especially if they are older (>7 years) and have a high CIBDAI score (>9). Dogs with elevated CRP levels also require medical attention. Cyclosporine therapy appears to have poor efficacy in Shiba dogs with CE. Early mortality is possible even if the initial treatment appears to be efficacious. Furthermore, cases with shorter duration (<3 months) to a response to initial treatment may have a shorter survival duration. Although dogs that survive for >6 months may remain alive after 1 year, approximately half die due to a relapse of the signs, and therefore Shiba dogs with CE require long-term follow-up.

### Ethical approval

Written informed consent was obtained from the owners for publication of the case presentations and accompanying images. A copy of the written consent is available for review by the Editor-in-Chief of this journal.

## Abbreviations

CE: Chronic enteropathies; CIBDAI: Canine inflammatory bowel disease activity index; IBD: Inflammatory bowel disease; ARD: Antibiotic-responsive diarrhea; FRD: Food-responsive diarrhea; CBC: Complete blood cell; PCV: Packed cell volume; WBC: White blood cell; PLT: blood platelet; AT: Antithrombin; PT: Prothrombin time; APTT: Activated partial thromboplastin time; Fib: Fibrinogen; BUN: Blood urea nitrogen; CRE: creatinine; ALP: alkaline phosphatase; ALT: alanine aminotransferase; Ca: Calcium; IP: Inorganic phosphorus; Glu: Glucose; Na: Sodium; K: Potassium; Cl: chloride; ALB: Albumin; Glob: Globulin; T. Chol: Total cholesterol; TP: Total protein; CRP: C-reactive protein; OR: Odds ratios; ROC: Receiver operator characteristic; LPE: Lymphocytic-plasmacytic enteritis; ACVP: American College of Veterinary Pathologists; AUC: Area under the curve; CCECAI: Canine chronic enteropathy clinical activity index; WSAVA: World Small Animal Veterinary Association

## Competing interests

The authors declare that they have no competing interests.

## Authors’ contributions

HO, TS, and TW participated in the design of the study. HO and YY performed the statistical analyses and YK carried out the histopathological analyses. All authors contributed to the interpretation of the data and to the drafting and revising of the manuscript. All authors read and approved the final manuscript.
